# A Longitudinal Study of Epileptic Seizures in Alzheimer's Disease

**DOI:** 10.3389/fneur.2019.01266

**Published:** 2019-12-04

**Authors:** John Baker, Tina Libretto, William Henley, Adam Zeman

**Affiliations:** ^1^Cognitive and Behavioral Neurology, College of Medicine and Health, University of Exeter, Exeter, United Kingdom; ^2^NIHR Exeter Clinical Research Facility, Royal Devon and Exeter NHS Foundation Trust, Exeter, United Kingdom; ^3^College of Medicine and Health, University of Exeter, Exeter, United Kingdom

**Keywords:** epilepsy, Alzheimer's disease, dementia, prognosis, prevalence

## Abstract

The prevalence of epileptic seizures is increased in patients in the clinical stages of Alzheimer's disease (AD) when compared to age-matched cognitively normal populations. In previously reported work from the Presentation of Epileptic Seizures in Dementia (PrESIDe) study, we identified a clinical suspicion of epilepsy in between 12.75 and 28.43% of patients with AD recruited from a memory clinic. EEGs were not performed in this study. Patients with epilepsy performed similarly to patients without epilepsy on cognitive testing at the time of recruitment but were more impaired on two measures of everyday functioning [Cambridge Behavioral Inventory—Revised and Clinical Dementia Rating (CBI-R and CDR)]. On repeated testing in this 12-month follow-up study, patients in whom a suspicion of epilepsy was identified performed significantly worse on cognitive function testing (*p* = 0.028) in addition to maintaining a difference on the informant questionnaires (CBI-R *p* < 0.001, CDR *p* = 0.020). These findings suggest that seizures in this population could be a marker of a more rapid decline and worse prognosis.

## Introduction

The prevalence of epileptic seizures is increased in patients in the clinical stages of Alzheimer's disease (AD) when compared to age-matched cognitively normal populations (1–3). However, the extent of this increased risk remains disputed (4–6). Whilst some studies have identified a risk which is similar to, or only slightly greater than the general population (7, 8); other studies have identified epileptic seizures in over 50% of AD patients (9, 10). There are several potential explanation for this divergence, including the means of data collection [retrospective (11) vs. prospective (12)], the populations being studied [new diagnosis (13) vs. advanced disease (10)] and the use of supplementary tests [electroencephalography and magnetoencephalography (14, 15)]. In the Presentation of Epileptic Seizures in Dementia (PrESIDe), 144 patients were recruited from a regional memory clinic. Participants were interviewed in their own home in the company of someone who knows them well (an informant). Of this group, 102/144 (70.83%) were diagnosed with AD using recognized diagnostic criteria (16, 17). Participants were divided into three groups, based on a structured interview (Appendix 1) administered to the informant, designed to elicit whether any epileptic seizures had occurred previously: epilepsy probable (E-Pr), epilepsy possible (E-Po), and no clinical evidence of epilepsy (NCEE) (Table 1). Using this method we identified a clinical suspicion of epilepsy in 29/102 (28.43%). This included 13/102 (12.75%) in whom it was felt that epilepsy was probable (E-Pr) and 16/102 (15.69%) in whom it was felt that epilepsy was possible (E-Po). These findings are comparable to those of recent studies in this field (14, 15, 18, 19). However, the long-term sequela of epilepsy in this population remain unclear. Does the presence of epileptic seizures affect the prognosis in these patients, and if so what is the nature and extent of this effect?

**Table 1:** Seizure group criteria.

Epilepsy probable	At least 2 stereotyped episodes suggestive of epilepsy witnessed by a reliable informant
Epilepsy possible	Single witnessed episode suggestive of epilepsy, or at least 2 episodes but not both reliably witnessed
No clinical evidence of epilepsy	No suspicious episodes reported by patient or informant
Seizure features: altered responsiveness, speech/behavioral arrest, oral/pharyngeal automatism, olfactory/gustatory aura, involuntary movements suggesting focal motor seizure, other sensory phenomena (including hallucination), amnesia on waking	

The aim of this study is to answer these questions through a 12-month follow-up assessment of patients initially recruited to the PrESIDe study. Our goal was to compare the rate of cognitive decline in E-Pr, E-Po, and NCEE participants. The initial findings of the PrESIDe study did not identify a significant difference in cognitive performance between these groups at the time of their initial memory clinic assessment or at the time of their recruitment in to the study. However, there was a difference in scores on informant completed questionnaires [Cambridge Behavioral Inventory—Revised and Clinical Dementia Rating (CBI-R and CDR)] which suggested increased difficulty in completing activities of daily living (ADLs) and greater care requirements in patients with dementia who also experienced epileptic seizures.

## Methods

We recruited patients to the PrESIDe study as outlined previously (20). Eleven months after their initial study assessment, participants were contacted via a letter to remind them of the study and to outline a plan to review them again. Letters were followed by a telephone call to schedule a follow-up visit. These interviews were to be performed 12 months (±2 weeks) after their initial visit, where possible in the same location, at the same time of day and in the presence of the same informant who was in attendance for the initial interview.

Assessments consisted of a brief interview to identify whether any further episodes suggestive of epilepsy had occurred, or if none had been identified at the time of the first interview, whether this had changed; as well as brief questioning to update information on medical history (any changes to medication, recent illnesses/surgery, etc.) over the intervening 12-months. Subsequently, cognitive testing was repeated using the Addenbrooke's Cognitive Examination—Version III (ACE-III) (Appendix 2). At the same time, the informant was asked to complete the same two questionnaires (CDR and CBI-R) in order to compare these with those previously completed.

Between-group analysis of demographic features, cognitive test performance and informant completed questionnaire scores was performed using independent sample *t*-tests and chi-square testing. Statistical significance was judged as any *p*-value < 0.05. Adjustments for multiple comparisons were not made as part of the exploratory analysis of these data. IBM SPSS statistics 22.0 and STATA were used to perform data analysis.

Ethical approval for this project was awarded through the Integrated Research Application System (IRAS) and provided by the London—Bromley Research Ethics Committee.

## Results

One hundred and two patients with AD were assessed and included in the initial study. Between the initial visit and the 11-month mail-out 6 patients had died. From this remaining sample of 96, the research team was unable to contact 17 patients and 7 patients declined further assessment. This resulted in a total of 72 (70.6%) participants receiving a 12-month follow-up assessment.

The demographic features of the group (*n* = 72) are summarized in Table 2 alongside the features of the total group seen at initial assessment (*n* = 102) for comparison. The participants seen for follow-up assessment did not differ significantly from the total group seen at baseline in terms of age, gender or ACE-III total score at initial interview. Of these 72 participants, 13 were classified E-Pr, 14 as E-Po [and therefore 27 patient in a combined epilepsy (COMB) group] and 45 as NCEE.

**Table 2:** Comparing baseline characteristics of total PrESIDe group with group seen for 12-month follow-up.

	**Initial PrESIDe group (*N* = 102)**	**12-month follow-up group (*N* = 72)**	
Age at baseline (mean, SD)	78.53, 6.47	78.69, 6.77	*P* = 0.880
Gender (M:F)	51:51	37:35	*P* = 0.857
ACE-III (at memory clinic)	74.53, 10.53	76.77, 9.25	*P* = 0.157
ACE-III (at baseline visit)	71.59, 10.86	73.82, 9.92	*P* = 0.169
Seizure diagnosis:			
Probable (E-Pr)	13 (12.75%)	13 (18.06%)	*P* = 0.335
Possible (E-Po)	16 (15.69%)	14 (19.44%)	*P* = 0.520
No clinical Evidence of Epilepsy (NCEE)	73 (71.57%)	45 (62.50%)	*P* = 0.209

### Cognitive Function

**Figure 1:**
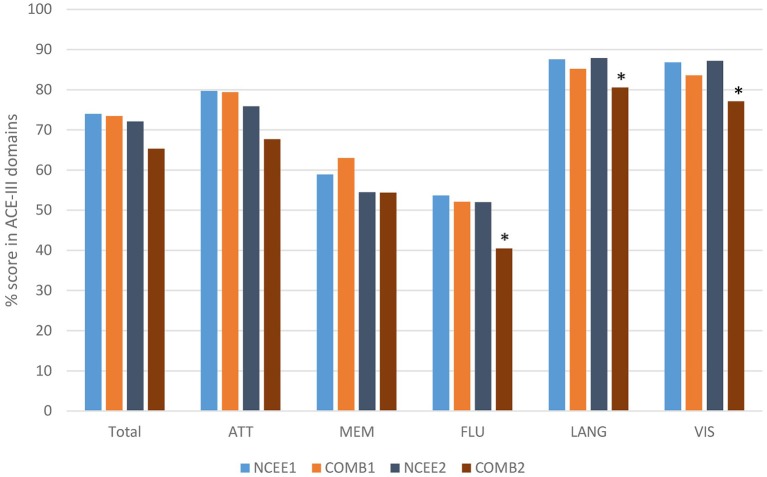
Comparison of ACE-III total score and sub-domain scores at baseline and 12-month follow-up (COMB vs. NCEE), ^*^indicates significant difference (*p* < 0.05) when compared to NCEE.

At the 12-month follow-up interview the total ACE-III score in the combined group was significantly lower than in the NCEE group (*p* = 0.028) (Table 3). The ACE-III score in the E-Po group was also significantly lower than the NCEE group at the 12-month follow-up (*p* = 0.030), whereas the E-Pr group was not significantly different to the NCEE group (*p* = 0.177). The size of the decline between PrESIDE baseline and 12-month follow-up was significantly greater in the E-Pr, E-Po, and COMB groups when compared to NCEE. The E-Po, E-Pr, and COMB groups showed a decline in all domains of the ACE-III test, with the largest declines seen in the attention, and fluency domains (Table 4). The declines in all domains in the COMB group were larger than those of the NCEE group (Figure 1).

**Table 3:** ACE-III test scores (mean and S.D) in participants with AD at different time points, with subjects categorized by suspicion of epilepsy, bold figures indicate significant difference (*p* < 0.05) when compared to NCEE group.

	**Memory clinic**	**PrESIDE baseline**	**PrESIDE 12-month**	**Change baseline to 12/12**
Total (*n* = 72)	76.79 (9.25)	73.82 (9.92)	69.56 (12.79)	4.26 (5.81)
NCEE (*n* = 45)	77.6 (9.7)	74.02 (9.83)	72.11 (11.51)	1.91 (3.69)
E-Po (*n* = 14)	75 (7.86)	71.79 (10.12)	**63.71 (14.82)**	**8.07 (7.62)**
	*P* = 0.365	*P* = 0.465	***P*** **=** **0.030**	***P*** **<** **0.001**
E-Pr (*n* = 13)	75.77 (9.31)	75.31 (10.44)	67 (13.14)	**8.31 (5.62)**
	*P* = 0.548	*P* = 0.683	*P* = 0.1773	***P*** **<** **0.001**
Comb (*n* = 27)	75.38 (8.45)	73.48 (9.31)	**65.3 (13.87)**	**8.19 (6.6)**
	*P* = 0.328	*P* = 0.819	***P*** **=** **0.028**	***P*** **<** **0.001**

**Table 4:** ACE-III domain scores in AD participants at PrESIDe baseline and 12-month follow-up, bold figures indicate significant difference (*p* < 0.05) when compared to NCEE group.

	**Att**	**Att (2)**	**±**	**Mem**	**Mem (2)**	**±**	**Flu**	**Flu (2)**	**±**	**Lang**	**Lang (2)**	**±**	**Vis**	**Vis (2)**	**±**
Total	79.6	72.8	−6.8	60.5	54.4	−6	53.1	47.6	−5.5	86.7	85.2	−1.5	85.6	83.4	−2.2
NCEE	79.7	75.9	−3.8	58.9	54.5	−4.5	53.7	52	−1.8	87.6	87.9	0.3	86.8	87.2	0.4
E–Po	76.6	67.1	−9.5	63.5	53.3	−10.2	44.4	**36.7**	−7.7	84.3	**79.7**	−4.7	83.5	**74.6**	−8.9
	*P* = 0.524	*P* = 0.125		*P* = 0.384	*P* = 0.832		*P* = 0.103	***P*** **=** **0.010**		*P* = 0.307	***P*** **=** **0.028**		*P* = 0.481	***P*** **=** **0.016**	
E–Pr	82.5	68.4	−14.1	62.4	55.6	−6.8	60.4	44.5	−15.9	86.1	81.7	−4.4	83.7	79.8	−3.8
	*P* = 0.548	*P* = 0.167		*P* = 0.516	*P* = 0.845		*P* = 0.240	*P* = 0.209		*P* = 0.659	*P* = 0.146		*P* = 0.514	*P* = 0.147	
Comb	79.4	67.7	−11.7	63	54.4	−8.5	52.1	**40.5**	−11.6	85.2	**80.6**	−4.6	83.6	**77.1**	−6.5
	*P* = 0.938	*P* = 0.074		*P* = 0.325	*P* = 0.982		*P* = 0.720	***P*** **=** **0.014**		*P* = 0.350	***P*** **=** **0.026**		*P* = 0.394	***P*** **=** **0.016**	

### Informant Questionnaires

Informant completed questionnaires highlighted significant differences between the epilepsy and non-epilepsy groups at both time points (Table 5). The CBI-R scores were significantly greater for the E-Po, E-Pr, and combined groups at the 12-month interval [*p* < 0.001 (E-Po), *p* = 0.02 (E-Pr), *p* < 0.001 (COMB)]. The CDR-SOB (sum of boxes) was significantly greater in the E-Pr (*p* = 0.019) and combined groups (*p* = 0.020) at the time of their baseline assessments and also at the time of the follow-up assessments [*p* = 0.036 (E-Pr), *p* = 0.05 (COMB)]. On this measure there was no significant difference between the NCEE and the E-Po group at either time point [*p* = 0.179 (baseline), *p* = 0.294 (12-month)]. The largest overall change in CDR-SOB was seen in the E-Pr group (1.21 points) and the smallest change was seen in the NCEE (0.93 points).

**Table 5:** Changes in CDR and CBI-R scores (mean and S.D) in AD participants between baseline interview and 12-month follow-up, bold figures indicate significant difference (*p* < 0.05) when compared to NCEE group.

	**CDR baseline**	**CDR 12-month**	**Change**	**CBI-R baseline**	**CBI-R 12-month**	**Change**
Total	4.20 (2.69)	5.20 (3.53)	+1.00	38.77 (22.68)	49.16 (25.26)	+10.39
NCEE	3.62 (2.46)	4.55 (3.26)	+0.93	32.69 (20.47)	40.03 (23.78)	+7.34
E-Po	4.67 (2.71)	5.67 (4.05)	+1.00	**55.00 (25.61)**	**72.00 (28.00)**	+17.00
	*P* = 0.179	*P* = 0.294		***P*** **=** **0.001**	***P*** **<** **0.001**	
E-Pr	**5.58 (2.98)**	**6.79 (3.52)**	+1.21	43.67 (19.74)	**57.92 (23.52)**	+14.25
	***P*** **=** **0.019**	***P*** **=** **0.364**		*P* = 0.092	***P*** **=** **0.020**	
Comb	**5.13 (2.83)**	**6.23 (3.76)**	+1.10	**49.09 (22.95)**	**64.65 (26.17)**	+15.56
	***P*** **=** **0.020**	***P*** **=** **0.049**		***P*** **=** **0.002**	***P*** **<** **0.001**	

On the CDR, the greatest differences between the combined E-Pr and E-Po group and the NCEE group were in the judgment and problem solving domain (0.56 points) and the personal care domain (0.47 points). The smallest differences were in the memory (0.10 points) and the orientation (0.11 points) sections. In contrast, for the CBI-R the most instructive questions (difference > 1.0) were found to be Memory 5 (Forgets the names of objects and things), Memory 8 (Becomes confused or muddled in unusual surroundings), and Sleep 2 (Sleeps more by day than before). Sleep 2 demonstrated the largest difference between these groups using the CBI-R (1.60 points) (Table 6). At the time of their initial PrESIDe assessment, the COMB group performed significantly worse than the NCEE group on 7/10 CBI-R domains (skill, care, behavior, mood, belief, eating, sleep). At the 12-month time point, this increased to 9/10 domains (memory, skill, care, behavior, belief, eating, sleep, motor, motivation).

**Table 6:** Mean changes in CBI-R domain scores in AD participants between baseline and follow-up assessments (higher scores indicate greater impairment), bold figures indicate significant difference (*p* < 0.05) when compared to NCEE group.

	**Mem1**	**Mem2**	**Change**	**Skill1**	**Skill2**	**Change**	**Care1**	**Care2**	**Change**
NCEE	13.3	15.6	+2.3	3.8	5.3	+1.5	0.2	0.8	+0.6
E-Po	14.9	**21.0**	+6.1	**8.1**	**10.2**	+2.1	**2.8**	**3.5**	+0.7
	*P* = 0.391	***P*** **=** **0.008**		***P*** **=** **0.002**	***P*** **=** **0.004**		***P*** **<** **0.001**	***P*** **=** **0.002**	
E-Pr	16.3	**19.7**	+3.4	6.4	7.1	+0.8	**1.5**	**2.3**	+0.8
	*P* = 0.105	***P*** **=** **0.033**		*P* = 0.079	*P* = 0.262		***P*** **<** **0.001**	***P*** **=** **0.043**	
Comb	15.6	**20.3**	+4.7	**7.2**	**8.6**	+1.4	**2.1**	**2.9**	+0.8
	*P* = 0.114	***P*** **=** **0.003**		***P*** **=** **0.003**	***P*** **=** **0.014**		***P*** **<** **0.001**	***P*** **=** **0.002**	
	**Behav1**	**Behav2**	**Change**	**Mood1**	**Mood2**	**Change**	**Belief1**	**Belief2**	**Change**
NCEE	1.8	2.8	+1	2.3	2.6	+0.3	0.1	0.5	+0.4
E-Po	**5.6**	**5.6**	0	**4.9**	3.9	−1	**0.9**	**2.7**	+1.8
	***P*** **<** **0.001**	***P*** **=** **0.023**		***P*** **=** **0.001**	*P* = 0.110		***P*** **<** **0.001**	***P*** **<** **0.001**	
E-Pr	2.5	3.6	+1.1	**3.8**	3.7	−0.1	**0.6**	1.0	+0.4
	*P* = 0.394	*P* = 0.451		***P*** **=** **0.040**	*P* = 0.174		***P*** **=** **0.001**	*P* = 0.244	
Comb	**4.0**	**4.6**	+0.6	**4.3**	3.8	−0.5	**0.7**	**1.8**	+1.1
	***P*** **=** **0.004**	***P*** **=** **0.049**		***P*** **=** **0.001**	*P* = 0.062		***P*** **<** **0.001**	***P*** **=** **0.004**	
	**Eating1**	**Eating2**	**Change**	**Sleep1**	**Sleep2**	**Change**	**Motor1**	**Motor2**	**Change**
NCEE	1.5	1.6	+0.1	2.3	2.5	+0.2	3.4	3.0	−0.4
E-Po	1.7	**3.8**	+2.1	3.5	**5.4**	+1.9	4.4	**6.0**	+1.6
	*P* = 0.798	***P*** **=** **0.007**		*P* = 0.054	***P*** **<** **0.001**		*P* = 0.363	***P*** **=** **0.003**	
E-Pr	1.9	**4.2**	+2.3	3.0	**4.1**	+1.1	3.5	4.3	+0.8
	*P* = 0.618	***P*** **=** **0.007**		*P* = 0.273	***P*** **=** **0.017**		*P* = 0.933	*P* = 0.196	
Comb	1.8	**4.0**	+2.2	**3.3**	**4.7**	+1.4	3.9	**5.1**	+1.2
	*P* = 0.620	***P*** **=** **0.001**		***P*** **=** **0.043**	***P*** **<** **0.001**		*P* = 0.587	***P*** **=** **0.012**	
	**Motiv1**	**Motiv2**	**Change**						
NCEE	3.9	5.2	+1.3						
E-Po	**8.1**	**9.8**	+1.7						
	***P*** **=** **0.007**	***P*** **=** **0.011**							
E-Pr	4.3	8.2	+3.9						
	*P* = 0.751	*P* = 0.090							
Comb	6.1	**9.0**	+2.9						
	*P* = 0.061	***P*** **=** **0.007**							

### Further Seizures

Eight patients reported having further witnessed seizure events between their initial study visit and their 12-month follow-up visit. In 3 cases, this occurred in patients who had previously been classified as E-Po leading to their reclassification as E-Pr. In 1 case, this occurred in patients who had previously been classified as NCEE. For the purpose of their analysis here they have been included in their original group. No further generalized onset tonic-clonic seizures were reported. Most commonly seizures were focal non-motor onset events involving behavioral arrest, cognitive, or sensory features. 3/12 patients who experienced further seizures were described as having motor automatisms at onset.

### Decline in Patients on Anti-epileptic Medication vs. Those Not on Treatment

4/27 COMB patients had been taking an anti-epileptic medication between the time of their baseline and 12-month assessments. In these patients there was a smaller mean decline in ACE-III scores (−5.75) than in those not taking anti-epileptic medication (−8.67), which was not significant (*p* = 0.42).

### Dementia Treatments

25/72 patients diagnosed with AD in our cohort (34.7%), were taking a medication prescribed for the treatment of their dementia between their baseline and 12-month assessments (18 Donepezil, 5 Rivastigmine, 2 Memantine). Whilst both groups saw a decrease in their ACE-III scores, this decrease was greater in the patients taking medication than in those that were not [−5.36 (SD 6.74) vs. −3.68 (SD 5.24)]. However, this difference was not significant (*p* = 0.246).

## Discussion

### Cognitive Function

Patients with AD and suspected epileptic seizures exhibit an accelerated decline in cognitive function when compared to patients with AD in whom there is no clinical suspicion of epilepsy. This is demonstrated by a fall in the mean ACE-III score from 74.02 to 72.11 (1.91 points) in the NCEE group and from 73.48 to 65.30 (8.19 points) in the COMB groups. Whilst, the NCEE group had a higher ACE-III score at the time of their initial memory clinic appointment and their baseline PrESIDe assessment, the difference in scores between this group and the COMB group only became significant at the time of the 12-month follow-up assessments.

Through, a domain specific analysis of the ACE-III scores we have shown that a decrease in performance occurs in all domains. The largest decreases in the epilepsy groups were seen in the attention, fluency, and memory components of the test. The difference in the decline between the epilepsy and non-epilepsy groups was greatest in the attention and fluency elements of the test. Whilst, all patients with dementia exhibit a decrease in cognitive function over time, those with epilepsy decline in a manner which is both greater and involves more domains, leading to the significant difference across the ACE-III total score. This supports the view that patients with Alzheimer's disease who experience epileptic seizures demonstrate a larger, multi-domain, decline in cognitive function than those without seizures. This is in contrast to the NCEE group, in whom the largest decline was seen in the memory domain, with relative stability in the language and visuospatial domains, and smaller declines in the fluency and attention domains.

The main question raised by these results is whether epilepsy is a marker for a more severe form of disease in these patients, or whether epilepsy is a driver of this more rapid change. The progression of clinical symptoms in AD is associated with the spread of the amyloid-β (Aβ) plaques and phosphorylated tau (p-tau) neurofibrillary tangles into different regions of the brain (21, 22). Those who experienced epileptic seizures demonstrate a more rapid decline across all domains. The reasons for this are not clear, although several studies have investigated the association between neuronal hyperexcitability and the spread of tau (23–25), suggesting that seizures can contribute to the spread of tau through both trans-neuronal (24, 26, 27) and trans-synaptic (28–30) means. Additionally, studies utilizing tau-PET have shown a direct correlation between the distribution of tau and cognitive impairment in patients with dementia (31, 32). It is possible that in patients with epileptic seizures the more rapid decline in cognitive function is related to an accelerated propagation of tau as a result of their epileptic seizures (33, 34). Conversely, it is also possible that some patients with AD experience a more aggressive form of this disease and that this phenotypic heterogeneity also give rise to epileptic seizures in these patients.

### Informant Questionnaires

The E-Pr group scored significantly higher on the CDR-SOB at the time of their initial study assessment. This difference persisted and even increased at the 12-month time-point. Whilst the difference in CDR-SOB at baseline between NCEE and the COMB groups was 1.51, at follow-up assessment this had increased to 1.68. Likewise, the CBI-R revealed roughly twice the decline in the COMB group by comparison with the NCEE group [7.34 points vs. 15.56 points (*p* = 0.034)]. These findings again suggest a more rapid accrual of deficits in these patients, identified by those nearest to them, and likely to increase their care requirements, and need for additional support.

### Anti-epileptic Treatment

We did not identify a significant difference in cognitive decline between COMB patients treated with antiepileptic medications and those that were not. It is possible that the lack of a significant difference is a result of the small size of these groups, or the limited duration of follow-up obtained. Other studies looking at the role of anti-epileptic medication in patients with dementia and animal models of dementia have reported conflicting outcomes (35–38). These may be explained by difference in patient selection, duration of therapy or drug dosage. Further research is required to define treatment guidelines for epilepsy occurring in the context of dementia.

Informal discussion with the clinicians referring into our study indicated that they were hesitant to prescribe anti-epileptic medications in patients with MCI and dementia. Correspondingly, only a small number of patients received treatment for their epilepsy in our cohort. This hesitation is partly due to concern about the possible cognitive side effects of these medications (38–40). Our study was not designed to interrogate the cognitive effects of anti-epileptic medication in these patients and further work is required investigate the potential risk and benefits of the wider use of these medications in patients with dementia. Multiple trials are currently underway on this topic (41).

### Dementia Treatments

No significant difference was identified in the rate of cognitive decline in patients with AD taking acetylcholinesterase inhibitors, or other medications licensed for the treatment of AD, vs. those that were not. In our cohort there was a difference between the ACE-III scores at baseline between these two groups that may explain this (70.75 in treated group vs. 75.35 in untreated group), although this was not significant (*p* = 0.063). There is however extensive evidence of the beneficial role of acetylcholinesterase inhibitors in Alzheimer's disease (42, 43). Our study was not designed to investigate the role of these medications and no effort was made to match those on these medications with those that were not treated at the time of their baseline assessment.

### E-Pr vs. E-Po

At all three time points in our study (memory clinic appointment, PrESIDe baseline assessment, 12-month follow-up) the two groups in whom epilepsy was suspected were similar. Whilst the E-Po group had a lower mean score on the ACE-III at all time points, the E-Pr group showed the largest decline from baseline PrESIDe assessment to 12-month follow-up. However, no significant differences were identified between them in terms of their ACE-III, CBI-R, or CDR scores at either baseline or 12-month assessments. This is not wholly surprising as the only clinical difference between them was whether or not they had had repeated witnessed episodes or not.

## Conclusion

The risk of epilepsy is increased in patients with Alzheimer's disease, and in this population a suspicion of epileptic seizures is associated with an accelerated rate of cognitive decline. This cognitive decline occurs across all cognitive domains measured by the ACE-III examination. The difference in the size of the decline was greatest in the attention and fluency domains of this test, suggesting that executive function is especially affected in this population.

Conventional understanding regarding epileptic seizures in patients with AD suggests that epilepsy occurs as a late-stage feature, and consequently treating seizures is unlikely to impact on the progression of disease or to result in any meaningful functional improvement for these patients. However, the findings of our study, in keeping with other recent reports (18, 44), suggest that epileptic seizures occur in patients at early clinical stages of AD and are associated with accelerated cognitive decline. This finding should encourage clinicians to identify patients who may have experienced epileptic seizures following the onset of their memory impairment and to consider anti-epileptic medication in these patients, where not contraindicated.

The limitations of our study include the relatively small size of our cohort, and limited duration of follow-up available. Whilst only a small number of participants (7/102) declined a follow-up interview, a larger number (17/102) could not be contacted. However, this rate of retention is in keeping with similar studies of this nature (45–47). We used clinical criteria to diagnose epilepsy. Further investigation would have been of interest. It is possible that the true incidence of epilepsy among patients with AD is even higher than we have reported. Several studies looking at the semiology of seizures in dementia, and AD in particular have recognized that seizures in this population are more likely to be focal in onset, often non-motor and rarely generalized tonic-clonic (18, 48, 49). Such subtle seizures are easily missed. Several recent studies have looked at the prevalence of subclinical epileptiform activity in these patients (14, 15, 19). In these studies prolonged EEG recording, or the use of more in-depth methods of analysis, such as magnetencephalography have been shown to identify abnormalities even in the absence of a clinical history of seizures.

Randomized controlled double-blind studies of the effects of anti-epileptic medications in appropriately selected patients with dementia and epilepsy are required in order to evaluate whether their use can improve the prognosis for patients who suffer from both conditions.

## Data Availability Statement

The datasets generated for this study are available on request to the corresponding author.

## Ethics Statement

The studies involving human participants were reviewed and approved by Integrated Research Application System (IRAS) and provided by the London—Bromley Research Ethics Committee. The patients/participants provided their written informed consent to participate in this study.

## Author Contributions

JB: design of study, ethical approval, data collection, analysis, drafting, and revision of paper. TL: recruitment of control group. WH: review of paper and statistical analysis. AZ: review of paper and Ph.D. supervision of JB.

### Conflict of Interest

The authors declare that the research was conducted in the absence of any commercial or financial relationships that could be construed as a potential conflict of interest.

## References

[1] Cheng CH Liu CJ Ou SM Yeh CM Chen TJ Lin YY. Incidence and risk of seizures in Alzheimer's disease: a nationwide population-based cohort study. Epilepsy Res. (2015) 115:63–6. 10.1016/j.eplepsyres.2015.05.009 26220378

[2] Cook M Baker N Lanes S Bullock R Wentworth C Arrighi HM. Incidence of stroke and seizure in Alzheimer's disease dementia. Age Ageing. (2015) 44:695–9. 10.1093/ageing/afv061 26008894

[3] Nicastro N Assal F Seeck M. From here to epilepsy: the risk of seizure in patients with Alzheimer's disease. Epileptic Disord. (2016) 18:1–12. 10.1684/epd.2016.0808 26907471

[4] Horvath A Szucs A Barcs G Noebels JL Kamondi A. Epileptic seizures in Alzheimer disease: a review. Alzheimer Dis Assoc Disord. (2016) 30:186–92. 10.1097/WAD.0000000000000134 26756385

[5] Abou-Khalil BW. How important is Alzheimer's disease as a risk factor for unprovoked seizures and epilepsy in the elderly? Epilepsy Curr. (2010) 10:36–7. 10.1111/j.1535-7511.2009.01347.x 20231919 PMC2836473

[6] Cretin B Philippi N Bousiges O Dibitonto L Sellal F Martin-Hunyadi C. Do we know how to diagnose epilepsy early in Alzheimer's disease? Rev Neurol. (2017) 173:374–80. 10.1016/j.neurol.2017.03.028 28501143

[7] Imfeld P Bodmer M Schuerch M Jick SS Meier CR. Seizures in patients with Alzheimer's disease or vascular dementia: a population-based nested case-control analysis. Epilepsia. (2013) 54:700–7. 10.1111/epi.12045 23215680

[8] Irizarry MC Jin S He F Emond JA Raman R Thomas RG. Incidence of new-onset seizures in mild to moderate Alzheimer disease. Arch Neurol. (2012) 69:368–72. 10.1001/archneurol.2011.830 22410444 PMC3622046

[9] Cabrejo L Guyant-Marechal L Laquerriere A Vercelletto M De la Fourniere F Thomas-Anterion C. Phenotype associated with APP duplication in five families. Brain. (2006) 129 (Pt 11):2966–76. 10.1093/brain/awl237 16959815

[10] Risse SC Lampe TH Bird TD Nochlin D Sumi SM Keenan T. Myoclonus, seizures, and paratonia in Alzheimer disease. Alzheimer Dis Assoc Disord. (1990) 4:217–25. 10.1097/00002093-199040400-00003 2264979

[11] Rao SC Dove G Cascino GD Petersen RC. Recurrent seizures in patients with dementia: frequency, seizure types, and treatment outcome. Epilepsy Behav. (2009) 14:118–20. 10.1016/j.yebeh.2008.08.012 18782632 PMC2875670

[12] Romanelli MF Morris JC Ashkin K Coben LA. Advanced Alzheimer's disease is a risk factor for late-onset seizures. Arch Neurol. (1990) 47:847–50. 10.1001/archneur.1990.00530080029006 2375689

[13] Scarmeas N Honig LS Choi H Cantero J Brandt J Blacker D. Seizures in Alzheimer disease: who, when, and how common? Arch Neurol. (2009) 66:992–7. 10.1001/archneurol.2009.130 19667221 PMC2768279

[14] Vossel KA Ranasinghe KG Beagle AJ Mizuiri D Honma SM Dowling AF. Incidence and impact of subclinical epileptiform activity in Alzheimer's disease. Ann Neurol. (2016) 80:858–70. 10.1002/ana.24794 27696483 PMC5177487

[15] Horvath A Szucs A Barcs G Kamondi A. Sleep EEG detects epileptiform activity in Alzheimer's disease with high sensitivity. J Alzheimer's Dis. (2017) 56:1175–83. 10.3233/JAD-160994 28128769

[16] McKhann GM Knopman DS Chertkow H Hyman BT Jack CR Jr Kawas CH. The diagnosis of dementia due to Alzheimer's disease: recommendations from the National Institute on Aging-Alzheimer's Association workgroups on diagnostic guidelines for Alzheimer's disease. Alzheimers Dement. (2011) 7:263–9. 10.1016/j.jalz.2011.03.005 21514250 PMC3312024

[17] Albert MS DeKosky ST Dickson D Dubois B Feldman HH Fox NC. The diagnosis of mild cognitive impairment due to Alzheimer's disease: recommendations from the National Institute on Aging-Alzheimer's Association workgroups on diagnostic guidelines for Alzheimer's disease. Alzheimers Dement. (2011) 7:270–9. 10.1016/j.jalz.2011.03.008 21514249 PMC3312027

[18] Vossel KA Beagle AJ Rabinovici GD Shu H Lee SE Naasan G. Seizures and epileptiform activity in the early stages of Alzheimer disease. JAMA Neurol. (2013) 70:1158–66. 10.1001/jamaneurol.2013.136 23835471 PMC4013391

[19] Horvath A Szucs A Hidasi Z Csukly G Barcs G Kamondi A. Prevalence, semiology, and risk factors of epilepsy in Alzheimer's disease: an ambulatory EEG study. J Alzheimers Dis. (2018) 63:1045–54. 10.3233/JAD-170925 29710705

[20] Baker J Zeman A Accelerated long-term forgetting in epilepsy-and beyond. In: Axmacher N Rasch B editors. Cognitive Neuroscience of Memory Consolidation. Cham: Springer International Publishing (2017). p. 401–17. 10.1007/978-3-319-45066-7_24

[21] Braak H Braak E. Staging of Alzheimer's disease-related neurofibrillary changes. Neurobiol Aging. (1995) 16:271–8; discussion 8–84. 10.1016/0197-4580(95)00021-6 7566337

[22] Braak H Del Tredici K. The preclinical phase of the pathological process underlying sporadic Alzheimer's disease. Brain. (2015) 138(Pt 10):2814–33. 10.1093/brain/awv236 26283673

[23] Lewis J Dickson DW. Propagation of tau pathology: hypotheses, discoveries, and yet unresolved questions from experimental and human brain studies. Acta Neuropathol. (2016) 131:27–48. 10.1007/s00401-015-1507-z 26576562

[24] Cope TE Rittman T Borchert RJ Jones PS Vatansever D Allinson K. Tau burden and the functional connectome in Alzheimer's disease and progressive supranuclear palsy. Brain. (2018) 141:550–67. 10.1093/brain/awx347 29293892 PMC5837359

[25] Kaufman SK Del Tredici K Thomas TL Braak H Diamond MI. Tau seeding activity begins in the transentorhinal/entorhinal regions and anticipates phospho-tau pathology in Alzheimer's disease and PART. Acta Neuropathol. (2018) 136:57–67. 10.1101/267724 29752551 PMC6015098

[26] Kim HR Lee P Seo SW Roh JH Oh M Oh JS. Comparison of amyloid beta and Tau spread models in Alzheimer's disease. Cereb Cortex. (2018) 29:4291–302. 10.1093/cercor/bhy311 30566579 PMC7963115

[27] Su JH Deng G Cotman CW. Transneuronal degeneration in the spread of Alzheimer's disease pathology: immunohistochemical evidence for the transmission of tau hyperphosphorylation. Neurobiol Dis. (1997) 4:365–75. 10.1006/nbdi.1997.0164 9440125

[28] Liu L Drouet V Wu JW Witter MP Small SA Clelland C. Trans-synaptic spread of tau pathology *in vivo*. PLoS ONE. (2012) 7:e31302. 10.1371/journal.pone.0031302 22312444 PMC3270029

[29] Wang Y Balaji V Kaniyappan S Kruger L Irsen S Tepper K. The release and trans-synaptic transmission of Tau via exosomes. Mol Neurodegen. (2017) 12:5. 10.1186/s13024-016-0143-y 28086931 PMC5237256

[30] Dujardin S Lecolle K Caillierez R Begard S Zommer N Lachaud C. Neuron-to-neuron wild-type Tau protein transfer through a trans-synaptic mechanism: relevance to sporadic tauopathies. Acta Neuropathol Commun. (2014) 2:14. 10.1186/2051-5960-2-14 24479894 PMC3922636

[31] Hanseeuw BJ Betensky RA Jacobs HIL Schultz AP Sepulcre J Becker JA Association of amyloid and Tau with cognition in preclinical alzheimer disease: a longitudinal study. JAMA Neurol. (2019) 76:915–24. 10.1001/jamaneurol.2019.1424 PMC6547132 31157827

[32] Ossenkoppele R Schonhaut DR Scholl M Lockhart SN Ayakta N Baker SL. Tau PET patterns mirror clinical and neuroanatomical variability in Alzheimer's disease. Brain. (2016) 139(Pt 5):1551–67. 10.1093/brain/aww027 26962052 PMC5006248

[33] Tai XY Koepp M Duncan JS Fox N Thompson P Baxendale S. Hyperphosphorylated tau in patients with refractory epilepsy correlates with cognitive decline: a study of temporal lobe resections. Brain. (2016) 139(Pt 9):2441–55. 10.1093/brain/aww187 27497924 PMC5926008

[34] Pooler AM Phillips EC Lau DH Noble W Hanger DP. Physiological release of endogenous tau is stimulated by neuronal activity. EMBO Rep. (2013) 14:389–94. 10.1038/embor.2013.15 23412472 PMC3615658

[35] Belcastro V Costa C Galletti F Pisani F Calabresi P Parnetti L. Levetiracetam monotherapy in Alzheimer patients with late-onset seizures: a prospective observational study. Eur J Neurol. (2007) 14:1176–8. 10.1111/j.1468-1331.2007.01907.x 17880574

[36] Sanchez PE Zhu L Verret L Vossel KA Orr AG Cirrito JR. Levetiracetam suppresses neuronal network dysfunction and reverses synaptic and cognitive deficits in an Alzheimer's disease model. Proc Natl Acad Sci USA. (2012) 109:E2895–903. 10.1073/pnas.1121081109 22869752 PMC3479491

[37] Nygaard HB Kaufman AC Sekine-Konno T Huh LL Going H Feldman SJ Brivaracetam, but not ethosuximide, reverses memory impairments in an Alzheimer's disease mouse model. Alzheimers Res Therapy. (2015) 7:25 10.1186/s13195-015-0110-9 PMC4419386 25945128

[38] Cumbo E Ligori LD. Levetiracetam, lamotrigine, and phenobarbital in patients with epileptic seizures and Alzheimer's disease. Epilepsy Behav. (2010) 17:461–6. 10.1016/j.yebeh.2010.01.015 20188634

[39] Eddy CM Rickards HE Cavanna AE. The cognitive impact of antiepileptic drugs. Ther Adv Neurol Disord. (2011) 4:385–407. 10.1177/1756285611417920 22164192 PMC3229254

[40] Ortinski P Meador KJ. Cognitive side effects of antiepileptic drugs. Epilepsy Behav. (2004) 5 (Suppl 1):S60–5. 10.1016/j.yebeh.2003.11.008 14725848

[41] ClinicalTrials.gov. Levetiracetam for Alzheimer's Disease-Associated Network Hyperexcitability. (2019). Available online at: https://ClinicalTrials.gov/show/NCT02002819

[42] Howard R McShane R Lindesay J Ritchie C Baldwin A Barber R. Donepezil and memantine for moderate-to-severe Alzheimer's disease. N Engl J Med. (2012) 366:893–903. 10.1056/NEJMoa1106668 22397651

[43] Lee JH Jeong SK Kim BC Park KW Dash A. Donepezil across the spectrum of Alzheimer's disease: dose optimization and clinical relevance. Acta Neurol Scand. (2015) 131:259–67. 10.1111/ane.12386 25690270

[44] Sarkis RA Dickerson BC Cole AJ Chemali ZN. Clinical and neurophysiologic characteristics of unprovoked seizures in patients diagnosed with dementia. J Neuropsychiatry Clin Neurosci. (2016) 28:56–61. 10.1176/appi.neuropsych.15060143 26404175

[45] Firth NC Primativo S Marinescu RV Shakespeare TJ Suarez-Gonzalez A Lehmann M. Longitudinal neuroanatomical and cognitive progression of posterior cortical atrophy. Brain. (2019) 142:2082–95. 10.1093/brain/awz136 31219516 PMC6598737

[46] Abshire M Dinglas VD Cajita MI Eakin MN Needham DM Himmelfarb CD. Participant retention practices in longitudinal clinical research studies with high retention rates. BMC Med Res Methodol. (2017) 17:30. 10.1186/s12874-017-0310-z 28219336 PMC5319074

[47] Eldholm RS Barca ML Persson K Knapskog AB Kersten H Engedal K. Progression of Alzheimer's disease: a longitudinal study in norwegian memory clinics. J Alzheimers Dis. (2018) 61:1221–32. 10.3233/JAD-170436 29254085

[48] Aller-Alvarez JS Menendez-Gonzalez M Ribacoba-Montero R Salvado M Vega V Suarez-Moro R. Myoclonic epilepsy in Down syndrome and Alzheimer disease. Neurologia. (2017) 32:69–73. 10.1016/j.nrleng.2014.12.019 25661268

[49] Bernardi S Scaldaferri N Vanacore N Trebbastoni A Francia A D'Amico A. Seizures in Alzheimer's disease: a retrospective study of a cohort of outpatients. Epileptic disord. (2010) 12:16–21. 10.1684/epd.2010.0290 20172846

