# Supplementary material for: A Longitudinal Study of Epileptic Seizures in Alzheimer's Disease
Source: Front Neurol. 2019 Dec 4;10:1266. doi: 10.3389/fneur.2019.01266 (PMC6904279; doi:10.3389/fneur.2019.01266)
Supplement: Supplementary file 1 [file Table_1.DOCX]

**Appendix1.**

| **Epileptic seizures in Dementia and MCI** | | | | | |
| --- | --- | --- | --- | --- | --- |
| **Data Capture - Initial interview** | | | | | |
|  |  |  |  |  |  |
| Interviewer |  |  | Interview date |  |  |
| Place of interview |  |  | Interview time |  |  |
|  |  |  |  |  |  |
| First name |  |  | DOB |  |  |
| Last name |  |  | Age when seen |  |  |
| Study ID |  |  |  |  |  |
|  |  |  |  |  |  |
| ***dementia details*** |  |  |  |  |  |
| ACE-III |  |  |  |  |  |
| Date seen in mem clinic |  |  | Mem clinic Diagnosis |  |  |
| Duration of symptoms |  |  | age at onset |  |  |
|  |  |  |  |  |  |
|  |  |  | Describe an example | | |
| memory difficulties |  | YES/ NO |  |  |  |
|  |  |  |  |  |  |
| visuospatial problems |  | YES/ NO |  |  |  |
|  |  |  |  |  |  |
| organisational problems |  | YES/ NO |  |  |  |
|  |  |  |  |  |  |
| Language problems |  | YES/ NO |  |  |  |
|  |  |  |  |  |  |
| arithmetical problems |  | YES/ NO |  |  |  |
|  |  |  |  |  |  |
| mood disturbance |  | YES/ NO |  |  |  |
|  |  |  |  |  |  |
| psychosis |  | YES/ NO |  |  |  |
|  |  |  |  |  |  |
| sleep disturbance |  | YES/ NO |  |  |  |
|  |  |  |  |  |  |
| behavioural disturbance |  | YES/ NO |  | | |
|  |  |  |  |  |  |
|  |  |  |  |  |  |
| example of memory problems |  |  |  |  |  |
|  |  |  |  |  |  |
|  |  |  |  |  |  |
|  |  |  |  |  |  |
|  |  |  |  |  |  |
|  |  |  |  |  |  |
|  |  |  |  |  |  |
| Assessment of fluctuation |  |  |  |  |  |
| One day fluctuation |  |  |  |  |  |
|  |  |  |  |  |  |
|  |  |  |  |  |  |
|  |  |  |  | Study ID: | |
|  |  |  |  | Participant Initials: | |
| fluctuation |  | YES/ NO |  |  |  |
| fluctuation example |  |  |  |  |  |
|  |  |  |  |  |  |
|  |  |  |  |  |  |
|  |  |  |  |  |  |
| Loss of consciousness |  | YES/NO |  |  |  |
| LOC example |  |  |  |  |  |
|  |  |  |  |  |  |
|  |  |  |  |  |  |
|  |  |  |  |  |  |
|  |  |  |  |  |  |
| ***Seizure features. Please indicate Y or N*** | | |  |  |  |
|  |  |  |  |  |  |
| Generalised onset seizures |  | YES/ NO |  |  |  |
|  |  |  |  |  |  |
| focal onset seizures |  |  |  |  |  |
| Automatisms |  | YES/ NO |  |  |  |
|  |  |  |  |  |  |
| Olfactory / gustatory hallucinations |  | YES/ NO |  |  |  |
|  |  |  |  |  |  |
| Deja-vu |  | YES/ NO |  |  |  |
|  |  |  |  |  |  |
| Period of altered responsiveness |  | YES/ NO |  |  |  |
|  |  |  |  |  |  |
| amnesic episodes (on waking) |  | YES/ NO |  |  |  |
|  |  |  |  |  |  |
| amnesic episodes (at other times) |  | YES/ NO |  |  |  |
|  |  |  |  |  |  |
| Repetitive questioning |  | YES/ NO |  | | |
|  |  |  |  |  |  |
|  |  |  |  |  |  |
| Triggers |  | YES/ NO |  |  |  |
|  |  |  |  |  |  |
| Aura |  | YES/ NO |  | | |
|  |  |  |  |  |  |
|  |  |  |  |  |  |
| example seizure |  |  |  |  |  |
|  |  |  |  |  |  |
|  |  |  |  |  |  |
|  |  |  |  |  |  |
|  |  |  |  |  |  |
|  |  |  |  |  |  |
| ***Family history*** |  |  |  | Study ID: | |
| Fam Hx dementia |  | YES/ NO |  | participant Initials: | |
| Fam Hx Epilepsy |  | YES/ NO |  |  |  |
| Fam Hx other |  |  |  |  |  |
|  |  |  |  |  |  |
|  |  |  |  |  |  |
| Current medications |  |  |  |  |  |
|  |  |  |  |  |  |
|  |  |  |  |  |  |
| Past-medical Hx |  |  |  |  |  |
|  |  |  |  |  |  |
|  |  |  |  |  |  |
|  |  |  |  |  |  |
| Birth trauma / Anoxia |  | YES/ NO |  |  |  |
| birth trauma / anoxia details |  |  |  |  |  |
|  |  |  |  |  |  |
|  |  |  |  |  |  |
| Febrile seizures |  | YES/ NO |  |  |  |
| Febrile seizures details |  |  |  |  |  |
|  |  |  |  |  |  |
|  |  |  |  |  |  |
| Significant head injury |  | YES/ NO |  |  |  |
| Head injury details |  |  |  |  |  |
|  |  |  |  |  |  |
|  |  |  |  |  |  |
| Intracranial infection |  | YES/ NO |  |  |  |
| Intracranial infection details |  |  |  |  |  |
|  |  |  |  |  |  |
|  |  |  |  |  |  |
| Stroke |  | YES/NO |  |  |  |
| Stroke details |  |  |  |  |  |
|  |  |  |  |  |  |
|  |  |  |  |  |  |
| EEG |  | YES/ NO |  |  |  |
| EEG result |  |  |  |  |  |
|  |  |  |  |  |  |
| MRI |  | YES/ NO |  |  |  |
| MRI result |  |  |  |  |  |
|  |  |  |  |  |  |
| CT |  | YES/NO |  |  |  |
| CT Results |  |  |  |  |  |
|  |  |  |  |  |  |
