# Supplementary material for: A Longitudinal Study of Epileptic Seizures in Alzheimer's Disease
Source: Front Neurol. 2019 Dec 4;10:1266. doi: 10.3389/fneur.2019.01266 (PMC6904279; doi:10.3389/fneur.2019.01266)
Supplement: Supplementary file 2 [file Data_Sheet_1.PDF]

ADDENBROOKE'S COGNITIVE EXAMINATION – ACE-III

English Version A (2012)

Name:<br>Date of Birth:<br>Hospital No. or Address:	Date of testing: ___/___/___<br>Tester's name:______________________________<br>Age at leaving full-time education:_____________<br>Occupation: _______________________________<br>Handedness: _______________________________

A T T E N T I O N

➢ Ask: What is the	Day<br>________	Date<br>________	Month<br>__________	Year<br>__________	Season<br>__________	**Attention** [Score 0-5]
➢ Ask: Which	No./Floor<br>________	Street/Hospital<br>________	Town<br>__________	County<br>__________	Country<br>__________	**Attention** [Score 0-5]

A T T E N T I O N

- ➢ Tell: "I'm going to give you three words and I'd like you to repeat them after me: lemon, key and ball." After subject repeats, say "Try to remember them because I'm going to ask you later".
- ➢ Score *only* the first trial (repeat 3 times if necessary).
- ➢ Register number of trials: _____

Attention [Score 0-3]

A T T E N T I O N

- ➢ Ask the subject: "Could you take 7 away from 100? I'd like you to keep taking 7 away from each new number until I tell you to stop."
- ➢ If subject makes a mistake, do not stop them. Let the subject carry on and check subsequent answers (e.g., 93, 84, 77, 70, 63 – score 4).
- ➢ Stop after five subtractions (93, 86, 79, 72, 65): ____ ____ ____ ____ ____

Attention [Score 0-5]

M E M O R Y

- ➢ Ask: 'Which 3 words did I ask you to repeat and remember?' _______ ________ ________

Memory [Score 0-3]

F L U E N C Y

- ➢ **Letters**

Say: "I'm going to give you a letter of the alphabet and I'd like you to generate as many words as you can beginning with that letter, but not names of people or places. For example, if I give you the letter "C", you could give me words like "cat, cry, clock" and so on. But, you can't give me words like Catherine or Canada. Do you understand? Are you ready? You have one minute. The letter I want you to use is the letter "P".

Fluency [Score 0 – 7]

≥ 18	7
14-17	6
11-13	5
8-10	4
6-7	3
4-5	2
2-3	1
0-1	0
total	correct

- ➢ **Animals**

Say: "Now can you name as many animals as possible. It can begin with any letter."

Fluency [Score 0 – 7]

≥ 22	7
17-21	6
14-16	5
11-13	4
9-10	3
7-8	2
5-6	1
<5	0
total	correct

M E M O R Y

➢ Tell: "I'm going to give you a name and address and I'd like you to repeat the name and address after me. So you have a chance to learn, we'll be doing that 3 times. I'll ask you the name and address later."<br><br>Score only the third trial.				**Memory**<br>[Score 0 – 7]
	1st Trial	***2nd Trial***	*3rd Trial*	
Harry Barnes<br>73 Orchard Close<br>Kingsbridge<br>Devon	________ ________<br>____ ______ ______<br>______________<br>______________	________ ________<br>____ ______ ______<br>______________<br>______________	________ ________<br>____ ______ ______<br>______________<br>______________	

M E M O R Y

- ➢ Name of the current Prime Minister……………………………………………………………………………..
- ➢ Name of the woman who was Prime Minister ……………………………………………………………………
- ➢ Name of the USA president…………………………………………………………………………………………..
- ➢ Name of the USA president who was assassinated in the 1960s……………………………………………

Memory
[Score 0 – 4]

L A N G U A G E

- ➢ Place a pencil and a piece of paper in front of the subject. As a practice trial, ask the subject to "**Pick up the pencil and then the paper**." If incorrect, score 0 and do not continue further.
- ➢ If the subject is correct on the practice trial, continue with the following three commands below.
  - Ask the subject to "**Place the paper on top of the pencil**"
  - Ask the subject to "**Pick up the pencil but not the paper**"
  - Ask the subject to "**Pass me the pencil after touching the paper**"

Note: Place the pencil and paper in front of the subject before each command.

Language
[Score 0-3]

L A N G U A G E

- ➢ Ask the subject to write two (or more) complete sentences about his/her last holiday/weekend/Christmas. Write in complete sentences and do not use abbreviations.
  Give 1 point if there are two (or more) complete sentences about the one topic; and give another 1 point if grammar and spelling are correct.

Language
[Score 0-2]

L A N G U A G E

- ➢ Ask the subject to repeat: **'caterpillar'; 'eccentricity; 'unintelligible'; 'statistician'**

Score 2 if all are correct; score 1 if 3 are correct; and score 0 if 2 or less are correct.

Language
[Score 0-2]

L A N G U A G E

➢ Ask the subject to repeat: **‘All that glitters is not gold’**	**Language** [Score 0-1]
➢ Ask the subject to repeat: **‘A stitch in time saves nine’**	**Language** [Score 0-1]

L A N G U A G E

➢ Ask the subject to name the following pictures:

Language [Score 0-12]

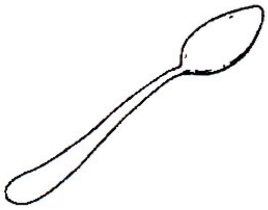
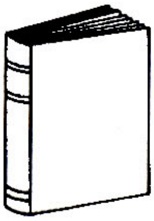
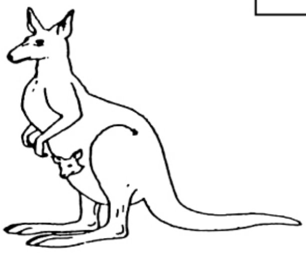
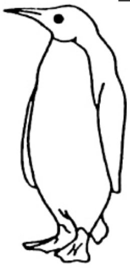
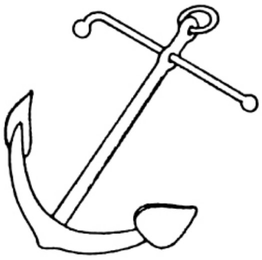
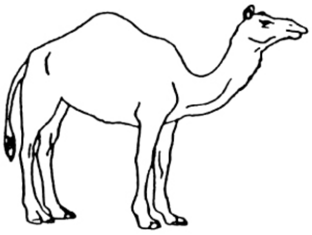
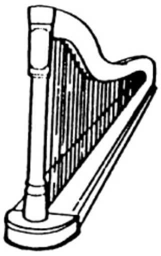
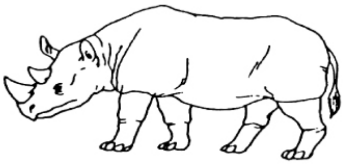
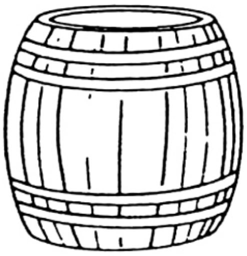
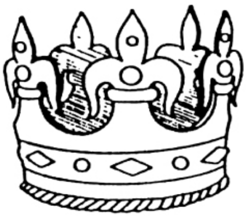
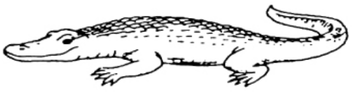
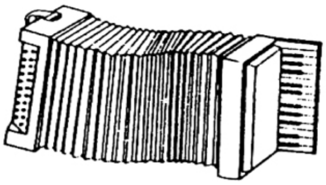

L A N G U A G E

➢ Using the pictures above, ask the subject to:

- Point to the one which is associated with the monarchy ……………………………………………..
- Point to the one which is a marsupial ……………………………………………..
- Point to the one which is found in the Antarctic ……………………………………………
- Point to the one which has a nautical connection ……………………………………………

Language [Score 0-4]

L A N G U A G E	
➢ Ask the subject to read the following words: (Score 1 only if all correct)<br><br>**sew**<br>**pint**<br>**soot**<br>**dough**<br>**height**	**Language**<br>[Score 0-1]

V I S U O S P A T I A L A B I L I T I E S	
➢ Infinity Diagram: Ask the subject to copy this diagram	**Visuospatial**<br>[Score 0-1]

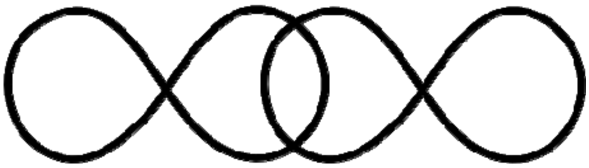

➢ Wire cube: Ask the subject to copy this drawing (for scoring, see instructions guide).	**Visuospatial**<br>[Score 0-2]

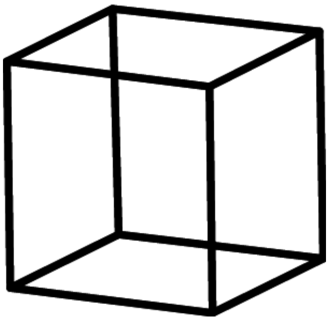

➢ Clock: Ask the subject to draw a clock face with numbers and the hands at ten past five. (For scoring see instruction guide: circle = 1, numbers = 2, hands = 2 if all correct).	**Visuospatial**<br>[Score 0-5]

V I S U O S P A T I A L   A B I L I T I E S

- Ask the subject to count the dots without pointing to them

Visuospatial
[Score 0-4]

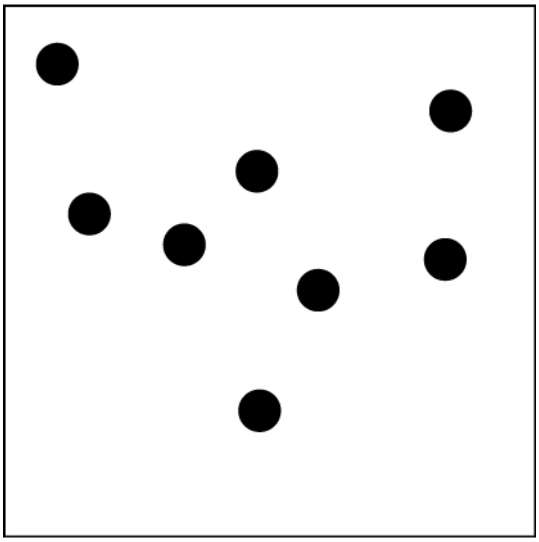

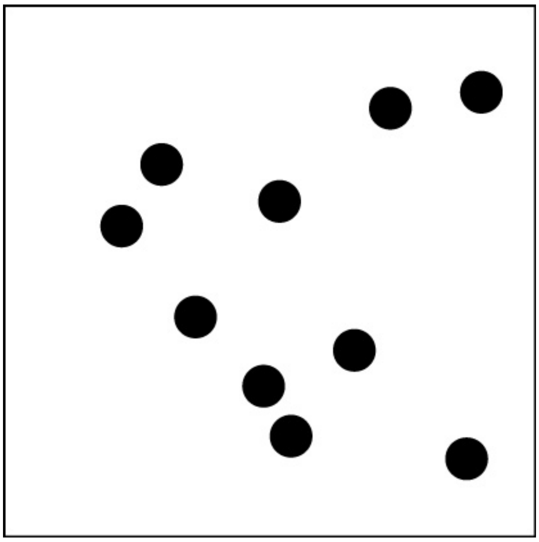

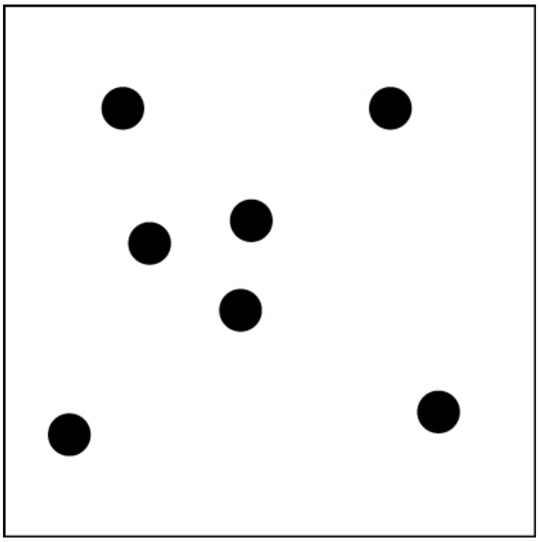

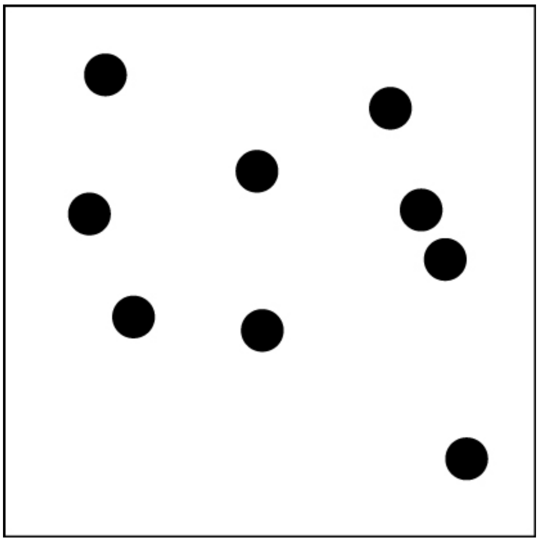

VISUOSPATIAL ABILITIES

➢ Ask the subject to identify the letters	**Visuospatial** [Score 0-4]

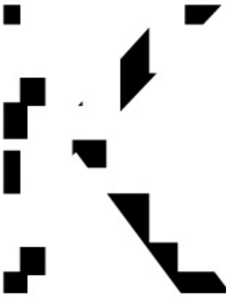

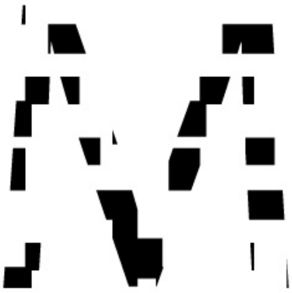

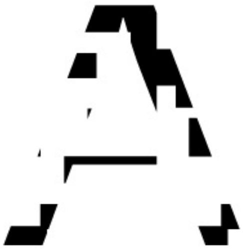

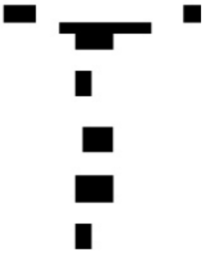

MEMORY

➢ Ask “Now tell me what you remember about that name and address we were repeating at the beginning”

Harry Barnes<br>73 Orchard Close<br>Kingsbridge<br>Devon	………………… ……………………………………<br>…… ……………………… ………………………<br>…………………………………………………………<br>…………………………………………………………	**Memory** [Score 0-7]

MEMORY

➢ This test should be done if the subject failed to recall one or more items above. It all items were recalled, skip the test and score 5. If only part was recalled start by ticking items recalled in the shadowed column on the right hand side; and then test not recalled items by telling the subject “ok, I’ll give you some hints: was the name X, Y or Z?” and so on. Each recognised item scores one point, which is added to the point gained by recalling.

Memory [Score 0-5]

Jerry Barnes		Harry Barnes		Harry Bradford		recalled	
37		73		76		recalled	
Orchard Place		Oak Close		Orchard Close		recalled	
Oakhampton		Kingsbridge		Dartington		recalled	
Devon		Dorset		Somerset		recalled	

SCORES

**TOTAL ACE-III SCORE**	/100
**Attention**	/18
**Memory**	/26
**Fluency**	/14
**Language**	/26
**Visuospatial**	/16
